# BRCA1：一种新的非小细胞肺癌放化疗疗效的预测基因

**DOI:** 10.3779/j.issn.1009-3419.2012.08.06

**Published:** 2012-08-20

**Authors:** 敏 李, 赛军 樊

**Affiliations:** 215123 苏州，苏州大学医学部放射医学与防护学院 School of Radiation Medicine and Protection, Medical College of Soochow University, Suzhou 215123, China

**Keywords:** 肺肿瘤, BRCA1, 放疗, 化疗, Lung neoplasms, Breast cancer susceptibility gene 1, Radiotherapy, Chemotherapy

## Abstract

非小细胞肺癌（non-small cell lung cancer, NSCLC）是世界范围内最常见的恶性肿瘤之一。放化疗是其治疗的主要方法，研究和确定临床上NSCLC放化疗敏感性预测和诊断的靶向分子和指标将有利于NSCLC患者的个体化治疗，以获得最佳治疗效果。本文就BRCA1作为NSCLC放化疗疗效的重要指标的研究现状做一综述，以期为临床NSCLC患者个体化放化疗提供参考。

## Abstract (en)

Non-small cell lung cancer (NSCLC) is one of the most common malignant tumors worldwide. Chemotherapy and radiotherapy are the main methods in therapy of NSCLC. The identification and characterization of predictive markers of radio- and chemo-sensitivity will be beneficial with customized treatment of NSCLC patients. This article will review the evidence regarding breast cancer susceptibility gene 1 (BRCA1) as a new predictive marker for chemotherapy and radiotherapy of NSCLC.

肺癌是世界上严重影响人类健康的主要恶性肿瘤之一，在最新的全球癌症统计中，肺癌的发病率和死亡率均居首位^[1, 2]^。而我国也是肺癌的高发国家，近十年肺癌已占据男、女性恶性肿瘤发病率的第一位和第二位，其发病率和死亡率一直呈上升趋势，排在癌症死因第一位，并呈现年轻化的趋势。而在所有癌症患者中，约80%-85%的肺癌患者为非小细胞肺癌（non-small cell lung cancer, NSCLC）患者。目前，由于缺乏有效的早期诊断方法，70%以上的NSCLC患者确诊时已处于晚期，无法对其进行有效的根治性手术切除，而只能通过化学药物治疗（化疗）或放射治疗（放疗）方法进行治疗。但由于个体之间的差异性和放化疗耐药的产生，术后辅助放化疗和晚期NSCLC患者的单独放化疗疗效不尽人意。肺癌放化疗治疗敏感性和耐药性的标志相关基因的研究和确定将可使肺癌治疗更加个体化，从而提高放化疗的疗效、减少不必要的副反应。累积的临床研究结果^[3]^表明，切除修复交叉互补基因1（excision repair cross complementation group 1, ERCC1）、核苷酸还原酶亚单位M1（ribonucleotide reductase subunit M1, RRM1）、胸苷合成酶（thymidylate synthetase, TS）、β-微管蛋白Ⅲ（β-tubulin Ⅲ)、DNA错配修复基因（主要包括human mutL homolog 1, hMLH1; human mutL homolog 1, hMSH2）、乳癌易感基因1（breast cancer susceptibility gene 1, BRCA1）、*K-ras*基因及表皮生长因子受体（epidermal growth factor receptor, EGFR）等分子标志物都可以作为预测NSCLC放化疗敏感性、指导NSCLC患者个体化治疗的指标。

BRCA1是一种具有遗传倾向的乳腺癌和卵巢癌的易感基因，是第一个被发现的遗传性乳腺癌易感基因^[4]^，并于1994年由Miki等^[5]^研究人员首次克隆获得。*BRCA1*基因定位于人类染色体17q21，全长为117 kb^[6]^，编码一个含有1, 863个氨基酸、分子量为220 kDa的蛋白质^[7]^。研究^[8]^发现，BRCA1通过其的转录调节、磷酸化、泛素化等功能，与Cyclin B、p21、GADD45、Chk2、Rb、Chk1、14-3-3等分子作用来调控细胞周期进程；而通过其与DNA损伤相关基因如ATM、ATR、RAD51、RAD50、MRE11、NBS1、BRCA2等相互作用参与包括同源性重组、非同源末端粘连、核苷酸切除修复在内的DNA损伤修复的复杂过程中^[9]^。BRCA1还可以通过与MAPK4、c-Jun、Fas、caspase-9等作用来调节细胞凋亡。BRCA1可与微管蛋白相互作用而定位在中心体上，这表明BRCA1在有丝分裂复制的染色体精确分离的过程中起着直接的作用，从而在细胞周期检验点的调控中起着重要的作用^[10]^。另外，BRCA1还可以通过调节血管形成素-1（angiopoietin-1, ANG1）、碱性成纤维细胞生长因子（basic fibroblast growth factor, GF）、雌激素受体（estrogen receptor, ER）等参与血管形成的调节，而ANG1与血管形成素-2（angiopoietin-2, ANG2）也是NSCLC的预后分子。

体外实验研究发现，上调BRCA1的表达可提高卵巢癌SKOV-3细胞的顺铂抗性^[11]^，在乳腺癌HBL100细胞中下调BRCA1的表达水平可增加对顺铂的敏感性，并可导致肿瘤细胞对抗微管药物的耐药^[12]^。而将功能型BRCA1质粒转入携带有畸变*BRCA1*基因的HCC1937乳腺癌细胞^[13]^同样可以使细胞对顺铂的敏感性明显降低，即抗性提高了20倍，而相反使细胞对包括紫杉醇和长春瑞滨在内的抗微管药物敏感性提高了1, 000倍-10, 000倍^[14]^。这些研究^[15]^结果表明BRCA1影响肿瘤细胞对不同化疗药物的敏感性可能存在双向调节的作用，所涉及的作用机制还需要做进一步的研究。

在2004年，Taron等^[16]^首次报道BRCA1 mRNA的表达水平与肺癌化疗（即术前化疗，临床多应用以顺铂为基础的化疗方案）抗性相关，之后，关于BRCA1表达与肺癌化疗疗效的临床性相关研究论文相继发表。本文就目前*BRCA1*基因与肺癌放化疗相关的研究结果做一简要的介绍，旨在为BRCA1作为一新的临床肺癌放化疗靶向分子、放化疗敏感性预测和治疗的预后指标提供依据。

## 1 BRCA1与肺癌患者化疗疗效和生存的关系

### 1.1 BRCA1作为化疗独立的预测及预后因素

铂类是NSCLC标准化疗治疗方案所必需的药物之一。目前治疗NSCLC的标准化疗方案主要是以顺铂为基础联合第三代化疗药物（吉西他滨、紫杉醇、长春瑞滨等）组成的化疗方案。铂类药物的作用机制主要是通过其与细胞核内的DNA相互结合，导致DNA链间或链内交联，产生DNA单链和双链的断裂和损伤，抑制肿瘤细胞的分裂，从而导致细胞的死亡。核苷酸切除修复（nucleotide excision repair, NER）在顺铂引起的DNA损伤修复过程中起着非常重要的作用，参与该修复途径的重要的基因和蛋白包括ERCC1、切除修复交叉互补基因2（exision repair cross-complementing group 2, ERCC2）、BRCA1等。

Taron等^[16]^完成了第一篇针对BRCA1对接受新辅助化疗的NSCLC患者化疗疗效及预后影响的回顾性临床研究，在对55例接受吉西他滨-顺铂联合（gemcitabine and cisplatin, GP）化疗的IIb期-Ⅲb期NSCLC患者的肺癌样本检测BRCA1 mRNA表达情况时发现，与BRCA1中度表达（化疗反应率为57.1%）和高表达患者（化疗反应率为58.3%）相比，BRCA1低水平表达的NSCLC患者对GP化疗的敏感程度有所增加（化疗反应率为66.7%）。BRCA1低水平表达患者的辅助化疗后手术完全切除率也高于BRCA1中度表达患者和高表达患者（分别为93.3%、78.6%、83.3%），并且BRCA1低水平表达患者也较中度和高表达患者采用肺叶切除术的治愈率更高，分别为73.3%、32.1%（*P*=0.005）和58.3%（*P*=0.2）。BRCA1低表达者的中位生存期（median survival time, MST）未达到，而BRCA1中度表达和高表达患者的MST分别为37.8个月和12.7个月。最后，其研究还发现，BRCA1低表达患者死亡率比高表达者死亡率低（HR=0.294, 95%CI: 0.05-0.83, *P*=0.026）。这些研究结果和随后的一些有关NSCLC患者辅助化疗的临床性研究结果^[17-23, 41, 42]^在不同程度上都表明，对接受含铂类辅助化疗治疗的患者而言，BRCA1低水平表达或阴性表达的患者化疗后的化疗有效率、无病生存期（progression free survival, PFS）、总生存期均优于BRCA1高表达或阳性表达的患者（表 1）。研究^[24, 25]^也发现BRCA1蛋白的Ser1613Gly位点变异与铂类药物化疗疗效相关，杂合型和携带Gly变异基因的肺癌患者化疗临床疗效及生存期都明显高于野生型*BRCA1*的肺癌患者。Shang等^[26]^的研究也显示，顺铂化疗耐药患者的BRCA1蛋白阳性水平明显高于化疗敏感的患者（分别为52.63%、26.83%，*P* < 0.05）。

**1:** BRCA1表达水平与非小细胞肺癌患者化疗疗效及预后相关性的临床研究 Summary of clinical study on the correlation of BRCA1 expression with prediction of chemotherapy response and prognosis in non-small cell lung cancer patients

Patients (*n*, phases)	Study type	Regimen	Sample type	BRCA1 expression		RR (%)	*P*	PFS (month)	*P*	MST (month)	*P*	1-yr(%)	2-yr(%)	3-yr(%)	Ref.
55, Ⅱb/Ⅲa/Ⅲb	Retrospective	GP/GC (neoadjuvant)	Tumor tissue	mRNA	Low	66.7				NR	0.01				Taron^[16]^
					Intermediate	57.1				37.8					
					High	58.3				12.7					
75, Ⅲb/Ⅳ	Retrospective	TP/NP	Tumor tissue	mRNA	Low	41	0.043			14	0.003	56.4	38.5		Yang^[17]^
					High	19.4				10		27.8	11.1		
136, Ⅲ/Ⅳ	Retrospective	GP/TP/TC/NP/TC	Tumor tissue	Protein	Negative	48.5	> 0.017	7	< 0.05	16	< 0.05				Zhang^[18]^
					Positive	37.1		5		10					
65, Ⅲ/Ⅳ	Retrospective	GP	Tumor tissue	Protein	Negative	45.83	< 0.05			13.2	< 0.05				Lu^[19]^
					Positive	21.95				8.4					
81, Ⅲb/Ⅳ	Retrospective	GP/NP/TP	Tumor tissue	Protein	Negative	56.4	0.01					54.8			Shan^[20]^
					Positive	31.0						30.8			
80, Ⅲa/Ⅲb/Ⅳ	Retrospective	GP/NP/TP	Tumor tissue	Protein	Negative	57.5	< 0.05								Mo^[21]^
					Positive	30.0									
81, Ⅲb-Ⅳ	Retrospective	NP/GP/TP	Tumor tissue		Negative	61.54	< 0.05			16.7	0.701				Shan^[22]^
					Positive	38.10				15.2					
63, Ⅰ/Ⅱ	Prospective	Adjuvant chemotherapy after resection	Tumor tissue	Protein	Negative									88.9	Zeng^[23]^
		Observation group after resection												34.1	
		Adjuvant chemotherapy after resection			Positive									69.9	
		Observation group after resection												77.1	
93, Ⅲa/Ⅲb/Ⅳ	Retrospective	GP/TP	Peripheral blood lyphocytes	DNA	Copy number, 0-1					14.57	0.0066				Kim^[24]^
					Copy number, 2					8.47					
124, Ⅲa/Ⅲb/Ⅳ	Retrospective	GP/TP/DP/NP/AP	Peripheral blood lyphocytes	DNA	Wild type A/A	1.00 (Ref.)	0.014								Su^[25]^
					Heterozygous type A/G	3.74	0.004								
					Mutant type G/G	1.64	0.503								
					Heterozygous type A/G+Mutant type G/G	3.25	0.006								
20, Ⅳ	Ex vivo	Cisplatin	Malignant effusions	mRNA	High	Low	0.014								Wang^[27]^
		Docetaxol			High	High	0.008								
96, Ⅲb/Ⅳ	Retrospective	DG	Tumor tissue	mRNA	Low	27.6	0.002	3.0	0.25	12	0.37				Boukovinas^[28]^
					Intermediate	13.8		3.6		8.5					
					High	58.6		5.5		12.7					
131, Ⅲb/Ⅳ	Retrospective	DG	Tumor tissue	mRNA	Low			3.5	0.013	11.1	0.458				Papadaki^[29]^
					High			6.2		10.2					
		DC			Low			6.8	0.874	11.2	0.912				
					High			6.1		11.1					
123, Ⅲ/Ⅳ	Prospective	GP	Tumor tissue	mRNA	Low	25		5		11			41.2		Rosell^[30]^
		DP			Intermediate	45.7		5		9			15.6		
		Docetaxol			High	41.9		8		11			0		
58, Ⅰb-ⅡB	Retrospective	Only resection	Tumor tissue	mRNA	Low			NR	0.04	NR	0.01				Rosell^[31]^
					High			22		29					
77, Ⅰ/Ⅱ/Ⅲa	Retrospective	XP(50)	Tumor tissue	Protein	Low					26.89±14.28	< 0.05				Sun^[32]^
					High					18.16±10.75					
32, Ⅰ/Ⅱ/Ⅲ	Retrospective	Only resection (6 cases), chemo- and radio- therapy after resection (6 cases), XP after resection (20 cases)	Tumor tissue	mRNA	Negative					17.4	0.01				Chen^[33]^
					Positive					NR					
156, Ⅳ	Retrospective	NP/DP/PI	Tumor tissue	Protein		20	0.62	6.1	0.67	15.4	0.06				Ota^[34]^
						27		5.4		8.7					
122, Ⅲb/Ⅳ	Retrospective	Unspecified	Tumor tissue	Protein	Negative	35.4	< 0.05		< 0.05	13.2		49.2			Gao^[35]^
					Positive	36.8				13.2		45.6			
RR: response rate; PFS: progression-free survival; MST: median survival time; 1-yr: 1-year survival rate; 2-yr: 2-year survival rate; 3-yr: 3-year survival rate; NR: not reached; GP: gemcitabine and cisplatin; GC: gemcitabine and carboplatin; TP: taxol and cisplatin; NP: vinorelbine and cisplatin; DP: docetaxol and cisplatin; DG: docetaxol and gemcitabine; AP: alimta and cisplatin; XP: cisplatin based combining therapy; PI: cisplatin and irinotecan.															

Wang等^[27]^的一项体外细胞研究表明，BRCA1对化疗敏感性的影响与化疗药物的种类有关，BRCA1表达水平与对顺铂的敏感性成负相关，但与对多西紫杉醇的敏感性呈正相关。Boukovinas等^[28]^通过检测102例采取吉西他滨与多西紫杉醇联合（gemcitabine and docetaxol, DG）一线化疗的晚期NSCLC患者肿瘤组织中的BRCA1 mRNA水平与化疗效果及预后的相关性，发现，BRCA1的表达与RRM1的表达呈明显的相关性。而在最终参与预测及预后研究的96例患者中，BRCA1高水平表达患者较BRCA1中、低水平表达患者的化疗反应率高（分别为58.6%、13.8%、27.6%，*P*=0.002）。而且，BRCA1高表达患者比BRCA1中和低表达患者的PFS（分别为5.5个月、3.6个月、3个月）和MST（分别为12.7个月、8.5个月、12个月）长，但无明显的统计学差异（*P*=0.25和*P*=0.37）。然而，BRCA1低水平表达的患者比BRCA1中、高水平表达患者进入二线化疗之前的时间相对较长（分别为6.6个月、2.0个月、2.4个月，*P*=0.004）。同样，另外一文献报道^[29]^对131例采用多西紫杉醇联合顺铂或吉西他滨治疗的Ⅲb期、Ⅳ期的NSCLC患者其BRCA1表达情况与化疗疗效及预后相关性分析，发现了同样的结果。在采用DG化疗组中，BRCA1高表达患者的PFS远长于BRCA1低表达患者（分别为6.2个月、3.5个月，*P*=0.013），但采用多西紫杉醇-顺铂联合（docetaxol and cisplatin, DP）的化疗患者则无明显差异。Rosell等^[30]^对111例晚期NSCLC患者BRCA1 mRNA进行前瞻性的分组检测，研究结果发现BRCA1低水平表达的患者接受GP方案，BRCA1中度表达的患者接受DP方案，而高水平表达的患者仅接受多西他赛单独治疗，结果三组患者的总生存期无明显差异，但2年生存率分别为41.2%、15.6%和0，提示BRCA1低表达者预后较好。并且根据BRCA1表达水平进行的个体化治疗疗效也高于意向治疗的疗效。这些实验结果表明尽管BRCA1高表达有利于多西紫杉醇等其它化疗药物的疗效，但不利于顺铂的治疗疗效。

其它一些研究^[31-33]^也表明，无论是只采用手术切除治疗还是术后予以采用顺铂的辅助化疗，BRCA1低表达或阴性者其预后生存都优于BRCA1高表达或阳性者。这些研究结果表明，BRCA1低表达者其预后较好。但也有临床研究^[34-39]^发现*BRCA1*基因与肺癌化疗敏感性及预后无相关性，甚至与上所描述的结果和结论正好相反。对此，可能需要更多的样本量相对较大的前瞻性临床研究来做进一步探讨。

### 1.2 BRCA1与其它生物标记分子联合应用

除了BRCA1以外，其它一些基因和蛋白如β-微管蛋白Ⅲ、ERCC1、ERCC1、EGFR等也都是目前肺癌治疗的重要预测及预后分子或治疗靶点。多项研究提示，单个基因的研究并不能准确判断预后，联合基因检测能提高预测的准确性。

#### 1.2.1 BRCA1与β-微管蛋白Ⅲ联合应用

Wan等^[40]^对采取紫杉醇-顺铂联合（taxol and cisplatin, TP）化疗的87例NSCLC患者根据BRCA1和β-微管蛋白Ⅲ表达高低分成4组：A组（BRCA1、β-微管蛋白Ⅲ低表达）、B组（BRCA1、β-微管蛋白Ⅲ高表达）、C组（BRCA1高表达、β-微管蛋白Ⅲ低表达）、D组（BRCA1低表达、β-微管蛋白Ⅲ高表达），观察近期疗效，结果显示，A组化疗反应率（response rate, RR）（60.7%）明显高于其它3组（34.8%, 9/19, 6/17）；而C组、D组RR虽高于B组，但差异无统计学意义。随访结果也显示，A组MST、PFS明显长于其它三组；而C组、D组MST、PFS虽长于B组，但差异无统计学意义，提示仅BRCA1、β-微管蛋白Ⅲ均低表达的NSCLC患者才有可能是TP化疗方案真正的靶向人群。

β-微管蛋白有7种异构体，其中仅有β-微管蛋白Ⅲ表达与微管蛋白结合类药物疗效密切相关。β-微管蛋白Ⅲ能降低微管蛋白的聚集速度，改变药物与微管蛋白二聚体连接部位，导致微管动力学特性的改变，对抗紫杉类药物导致的微管蛋白聚集，从而导致紫杉类药物的敏感性降低，产生耐药性。2011年的发表一篇*meta*分析研究^[41]^显示，β-微管蛋白Ⅲ低表达的晚期NSCLC患者对紫杉类化疗药疗效明显，其PFS、MST也明显高于β-微管蛋白Ⅲ高表达的患者，但在目前的研究中，β-微管蛋白Ⅲ表达水平与长春碱类药物疗效关系不一致，似乎其对长春碱类药物疗效的预测不如对紫杉类明显。

#### 1.2.2 *BRCA1*、*ERCC1*、*ERCC2*基因多态性检测联合应用

Su等^[25]^通过采用TaqMan探针法对124例接受含铂类药物化疗的晚期NSCLC患者的BRCA1 Ser1613Gly、ERCC1 Asn118Asn、ERCC2 Lys751Gln进行基因型分析，探讨核苷酸切除修复系统（nucleotide excision repair, NER）的3个重要基因*ERCC1*、*XPD*和*BRCA1*的单核苷酸多态性（single nucleotide polymorphisms, SNPs）与晚期NSCLC患者铂类药物化疗效果的相关性，结果发现，这三个基因多态存在一定的联合作用，携带变异等位基因（ERCC1 T、ERCC2 Gln和BRCA1 Gly）的数目越多，化疗临床受益率越高（*P*=0.036），提示联合分析多个基因的SNPs可能对指导化疗药物的选择更有帮助。Wen等^[42]^用免疫组化方法检测ERCC1、BRCA1在89例手术完全切除并行含铂辅助化疗的Ⅲa-N2期NSCLC中的表达，回顾性分析ERCC1和BRCA1的表达与临床、病理各因素及患者预后的关系。结果发现，ERCC1和BRCA1的表达与铂类化疗明显相关，表达阴性者在含铂化疗组有更长的无瘤生存期（*P* < 0.001, *P*=0.022）和总生存期（*P* < 0.001, *P*=0.009）。依据2个指标的表达水平分为4组：ERCC1和BRCA1表达均阴性；ERCC1表达阳性，BRCA1表达阴性；ERCC1表达阴性，BRCA1表达阳性；ERCC1和BRCA1表达均阳性。生存分析结果发现，ERCC1和BRCA1表达均为阴性时，患者的无瘤生存期及总生存期均优于其它组，*P* < 0.001；ERCC1和BRCA1表达均为阳性时，患者的无瘤生存期及总生存期最短。Papadaki等^[43]^的临床研究结果得出了与此一致的结论。

*ERCC1*和*ERCC2*是核苷酸切除修复过程中密切相关的两个基因，*ERCC2*基因产物具有5’-3’DNA解旋酶活性，可以解开损伤部位的DNA双链；然后ERCC1与着色性干皮病基因F（xeroderma pigmentosum goup F, XPF）形成的异源二聚体（具有5’核酸内切酶活性）可以在损伤位点15个-24个核苷酸处切开DNA单链，去除损伤的DNA片段。最新发表的一篇关于*ERCC1*、*ERCC2*基因多态性与接受含铂化疗的NSCLC患者的预测价值的*meta*分析综述性研究^[44]^总结发现，ERCC1 C118T基因多态性与NSCLC对顺铂类化疗的敏感性有明显的关系。

#### 1.2.3 BRCA1与RRM1表达水平检测联合应用

RRM1是核苷酸还原酶（ribonucleotide reductase, RR）亚单位M1，核糖核苷酸还原成脱氧核糖核苷酸是DNA合成的重要步骤，吉西他滨是核苷酸还原酶的竞争性抑制剂，通过竞争影响DNA的合成而起到杀伤肿瘤的目的。目前已有研究^[45]^发现，RRM1低表达或阴性表达预示着采用含吉西他滨化疗方案晚期NSCLC患者更高的RR和较好的预后。Boukovinas等^[28]^在研究中将采用吉西他滨-多西紫杉醇一线化疗的患者根据其PFS长短与BRCA1、RRM1表达将患者分为3组。结果发现，24例患者处于低风险组（中表达BRCA1+低表达RRM1，高表达BRCA1+低表达RRM1，高表达BRCA1+中表达RRM1），42例患者处于中风险组（低表达BRCA1+低表达RRM1，中表达BRCA1+高表达RRM1，高表达BRCA1+高表达RRM1），30例患者处于高风险组（低表达BRCA1+中表达RRM1，中表达BRCA1+中表达RRM1，低表达BRCA1+高表达RRM1），即中、高水平表达BRCA1，低、中水平表达RRM1对DG化疗方案预后较好，可能其中BRCA1预测了多西紫杉醇的疗效，而RRM1预测了吉西他滨的疗效。

#### 1.2.4 ERCC1、BRCA1和RRM1蛋白联合表达与化疗有效率的关系

Mo等^[21]^的研究表明，当将BRCA1作为单独指标时，BRCA1阴性表达组40例NSCLC患者，化疗有效者23例，含铂化疗反应率为57.5%，而BRCA1阳性表达组40例患者中，化疗有效患者则只有12例，有效率为30%（*P* < 0.05）。BRCA1、ERCC1、RRM1三个指标均阴性15例患者中，化疗有效10例，有效率则为66.7%，而在3个指标均阳性的22例患者，化疗有效2例，有效率为31.8%。显然，ERCC1、BRCA1和RRM1三个指标均阴性患者的化疗有效率明显高于均阳性者（χ^2^=4.316, *P* < 0.05）。Sun等^[32]^的研究发现，77例NSCLC组织中，BRCA1和ERCC1、BRCA1和EGFR之间的表达呈正相关（*rs*=0.823, *P* < 0.01; *rs*=0.583, *P* < 0.01）。Gu等^[46]^的研究也发现，110例NSCLC患者中，ERCC1及BRCA1的表达均为阳性者53例，均为阴性者16例，ERCC1与BRCA1表达强度间呈明显正相关（*r*=0.264, *P* < 0.01）。

#### 1.2.5 *EGFR*突变与BRCA1、RAP80表达水平检测联合应用

EGFR是目前肿瘤治疗的重要靶点。EGFR是一细胞膜表面的糖蛋白受体，为原癌基因*c-erbB1*编码表达的产物，并具有酪氨酸激酶活性。表皮生长因子受体酪氨酸激酶抑制剂吉非替尼与厄洛替尼是目前肺癌重要的分子靶向治疗药物，但临床研究发现，仅携带*EGFR*激活突变的患者应用EGFR-TKIs有着比较好的治疗反应。

Rosell等^[30]^对123例已转移的NSCLC患者根据其EGFR、BRCA1表达情况实行个体化化疗治疗，其中，携带*EGFR*突变的12例患者接受厄洛替尼治疗，其他未携带EGFR患者则根据BRCA1表达情况选择化疗方案：BRCA1低表达者采用吉非替尼-顺铂化疗，BRCA1中表达者采用DP化疗，BRCA1高表达者采用多西紫杉醇单独化疗。研究结果发现，EGFR突变患者、BRCA1低、中和高表达患者的化疗反应率分别为90%、25%、45.7%和41.9%。而意向治疗组（即随机治疗组），*EGFR*突变组、BRCA1低、中和高表达组患者的化疗反应率分别为75%、21.1%、40%和39.4%。*EGFR*突变组的生存中值未达到，但已超过了28个月，与BRCA1低、中和高表达组的患者生存中值10个月形成了对比。*EGFR*突变组患者的两年生存率为73.3%，BRCA1表达组所有患者的2年生存率为26.7%。在11例BRCA1与RAP80均低表达的患者中，其预后结果与*EGFR*突变组相持平，生存中值未达到，而无病生存期则为14个月。这些数据表明联合检测BRCA1与RAP80的表达水平可能比BRCA1与RAP80单独作为诊断指标对预测肺癌化疗更有意义。由于ERCC1、BRCA1表达情况与铂类药物敏感性相关，而RRM1表达水平与吉西他滨药物敏感性相联系，因而，我们认为，应用BRCA1、RRM1与ERCC1联合分析来预测患者对吉西他滨-顺铂联合化疗疗效与预后可能会更有价值。

## 2 BRCA1在肺癌及癌旁组织中的表达

Chen等^[33]^的研究发现，通过16例NSCLC癌组织和相应的16例癌旁组织的比较，BRCA1在肺癌组织中的表达明显高于癌旁组织，且BRCA1 mRNA表达量（相对于β-actin）也高于癌旁正常组织，分别为0.98×10^-3^和0.29×10^-3^（*P*=0.012）。Sun等^[32]^也获得了同样的研究结果。Yang等^[47]^的研究也显示，NSCLC肿瘤组织中BRCA1阳性率（60.87%, 70/115）明显高于癌旁组织的阳性率（15.38%, 10/65）。但Han等^[48]^的研究得出了相反的结论，BRCA1在NSCLC肺癌组织中的表达低于癌旁组织，表达阳性率分别为71.9%（23/32）和95.8%（23/24）。这些研究结果总结在表 2。BRCA1在肺癌组织和正常肺组织中的表达差异可能还需要样本量相对较大的临床性研究做进一步的研究。

**2:** BRCA1在NSCLC组织及癌旁组织中表达差异的研究 Expression of BRCA1 in NSCLC tissues and adjacent non-tumor tissues

BRCA1 expression	NSCLC tissue	Adjacent tissue	*P*	Reference
M (×10^3^)	0.98	0.29	0.012	Chen^[33]^
Positive rate	84.4% (65/77)	33.3% (2/6)	0.006	Sun^[32]^
Positive rate	60.9% (70/115)	15.4% (10/65)	< 0.05	Yang^[47]^
Positive rate	71.9% (23/32)	95.8% (23/24)	< 0.05	Han^[48]^
M: median (relative mRNA expression level of *β*-actin). NSCLC: non-small cell lung cancer.				

## 3 BRCA1与肺癌转移

目前有关BRCA1与肿瘤转移的研究，结论未达成统一。例如有些研究^[47, 49, 50]^表明BRCA1的表达情况与肺癌转移无关，但Han等^[48]^的研究比较13例无淋巴结转移和19例有淋巴结转移的NSCLC发现，BRCA1阳性表达在无淋巴结转移的肺癌组织中明显高于有淋巴结转移的肺癌组织，分别为76.9%和31.6%（*P* < 0.05）。

## 4 新辅助化疗对BRCA1表达的影响

肺癌的新辅助化疗是1981年SkarinFrei首先倡导的，其定义为在局部治疗前的细胞减量治疗，是手术与化疗相结合的另一种形式，与辅助化疗相比，新辅助化疗就是将全身治疗提前至局部治疗之前进行。近年来新辅助化疗在众多基础研究及临床实践中已经显示出了优越性，认为可以提高肿瘤切除率、无瘤生存率和总生存率。但新辅助化疗对术后化疗肿瘤耐药性的影响往往被忽视。而联合检测两者在肺癌新辅助化疗前后的表达情况目前仅发现1例报道。Gu等^[46]^通过免疫组化PV二步法检测110例NSCLC（50例术前行新辅助化疗，60例行常规手术而术前未经化疗）肿瘤组织中ERCC1和BRCA1的表达。结果发现，新辅助化疗组ERCC1和BRCA1总阳性表达率分别为68.00%和80.00%，单纯手术组为51.67%和70.00%，两者之间具有统计学差异（µ=-2.365和-3.011，*P* < 0.05）。这些研究表明新辅助化疗可以诱导NSCLC组织中ERCC1和BRCA1的表达，从而影响肿瘤对顺铂化疗的敏感性，在肿瘤化疗中应予重视和进一步的研究。因此，临床上应该严格选择新辅助化疗的适用对象，以免影响患者术后化疗的效果。同时，对应用新辅助化疗的患者，可通过检测ERCC1和BRCA1在新辅助化疗后的表达水平，了解肿瘤对铂类药物敏感性的情况，对新辅助化疗后两者明显升高的患者，术后辅助化疗可以选择非铂的化疗方案，以提高和明确化疗的疗效。

## 5 BRCA1与肺癌放射敏感性

有关BRCA1与肺癌放射敏感性的研究目前除了笔者所在的研究室最近发表了相关一篇论文外，国内外还未见报道。笔者所在的研究室的研究^[51]^发现，肺癌细胞的内源性BRCA1的表达水平与细胞的放射敏感性成负相关，BRCA1高表达的肺癌细胞放射敏感性比BRCA1低表达的肺癌细胞放射敏感性低。而且，采用人为的BRCA1表达质粒转染方法提高肺癌细胞中BRCA1表达水平可以降低细胞的放射敏感性，而采用siRNA转染方法降低BRCA1的表达水平则可以明显提高细胞的放射敏感性。而且，我们未发表的研究结果也证实在异体肺癌移植动物中，BRCA1高表达的移植肿瘤确实比BRCA1低表达的移植肿瘤的放射治疗疗效要差。这些研究说明BRCA1也可能是肺癌细胞放射治疗敏感性的重要指标，改变肺癌细胞中BRCA1的表达水平可以调节肺癌细胞的放射敏感性。

## 6 结论

综上所述，BRCA1可以作为肺癌放化疗疗效的重要临床诊断和预后指标，特别是结合其它的肺癌分子指标将为肺癌放化疗的个体化治疗方案的制定提供有效的参考。而且*BRCA1*基因治疗的研究和发展也毫无疑问为提高肺癌放化疗的疗效提供了一个新的辅助治疗手段。为此，许多相关的科学问题还需要我们做更深入的研究和探讨。例如：BRCA1的表达水平与肺癌化疗的疗效高低和预后是否与临床肺癌的类型有着密切的关系，而只是在NSCLC？如何采用模拟体内肿瘤生长的体外肺癌细胞模型明确改变BRCA1的表达水平对哪些化疗药物的疗效有着明显的影响？是否像体外实验结果那样，BRCA1的表达水平与肺癌放疗是否也存在一定的关系？BRCA1调节肺癌放化疗敏感性和疗效的作用作用机制何在？是否与BRCA1参与DNA损伤修复有关？等等。我们相信包括BRCA1在内的诊断靶分子和靶蛋白的研究深入和临床实际应用将为肺癌放化疗治疗疗效的提高提供新的基础和保障。
